# Full RAMIE vs Hybrid RAMIE: a retrospective study on outcomes evaluation and cost considerations

**DOI:** 10.1007/s13304-025-02180-7

**Published:** 2025-04-06

**Authors:** Elettra Ugliono, Fabrizio Rebecchi, Sara Salomone, Caterina Franco, Felice Borghi, Mario Morino

**Affiliations:** 1 General Surgery and Center for Minimally Invasive Surgery, Department of Surgical Sciences, University of Torino, Corso A.M. Dogliotti 14, 10126 Turin, Italy; 2 Oncologic Surgery Unit, Candiolo Cancer Institute, FPO-IRCCS, Candiolo, TO Italy

**Keywords:** Esophagectomy, Robot-assisted esophagectomy, Minimally invasive esophagectomy, Esophageal cancer

## Abstract

To compare the results of Minimally Invasive Esophagectomy performed with a Fully Robot-Assisted technique (F-RAMIE) and a Hybrid approach with laparoscopic abdominal phase (H-RAMIE). Multicentric retrospective analysis of patients who underwent F-RAMIE and H-RAMIE between 2018 and 2023. The primary endpoint was the rate of postoperative complications, secondary endpoints were clinical outcomes, oncological results and costs. Survival analyses were calculated according to the Kaplan–Meier method. The economic evaluation included costs related to operating room time, length of stay, surgical tools, and robotic system maintenance. A total of 100 patients from two experienced surgical centers were included: 64 H-RAMIE and 36 F-RAMIE. The two groups were comparable in baseline clinical conditions and staging. F-RAMIE was associated with longer operative time (434.7 ± 46.4 Vs. 477.3 ± 47.5 min, p < 0.001) and shorter length of Intensive Care Unit stay (1.1 ± 1.1 Vs. 2.3 ± 2.3 days, p = 0.002) than H-RAMIE. There were no significant differences in conversion rate, postoperative complications and length of stay. F-RAMIE demonstrated superior lymph node retrieval (43.8 ± 15.2 Vs. 22.4 ± 10.3, p < 0.001), but no differences in R0 resection rates. Overall survival and recurrences were comparable. Cost analysis revealed a slight economic advantage for F-RAMIE (20,556.3 ± 3,601.2 € Vs. 23,302.4 ± 5,894.5 € p = 0.012), mainly due to hospital stay-related cost (11,267.6 ± 5,912.8 € for H-RAMIE Vs. 8,360.3 ± 3,550.6 €, p = 0.007). F-RAMIE and H-RAMIE proved to be equally safe and effective in terms of postoperative complications and oncological outcomes.

## Introduction

Esophagectomy is the cornerstone of treatment for resectable esophageal cancer [1]. It is a technically complex procedure associated with considerable perioperative morbidity and a non-negligible mortality rate [2].

Over the years, minimally invasive laparo-thoracoscopic and robot-assisted techniques have gained popularity due to reduced surgical trauma and improved postoperative outcomes compared to traditional open esophagectomy [3–5].

The use of the robotic platform has demonstrated a disruptive effect in the thoracic phase of esophagectomy due to the technical advantages offered, such as three-dimensional vision and full dexterity, in performing a precise dissection around thoracic vital organs, without compromising oncological radicality [6, 7]. However, its role in the abdominal phase is less clear, especially when compared to minimally invasive laparoscopic approaches. In fact, there is limited evidence in the literature assessing the results of robot-assisted and laparoscopic abdominal phases of minimally invasive esophagectomy [8, 9].

This study aims to compare the outcomes of Robot-Assisted Minimally Invasive Esophagectomy (RAMIE) performed with a Fully Robotic (F-RAMIE) or a Hybrid laparoscopic abdominal approach (H-RAMIE) in terms of postoperative morbidity, oncological outcomes, and costs.

## Methods

We analyzed a prospectively collected multicentric database of patients who underwent F-RAMIE and H-RAMIE in two high-volume institutions in Northern Italy between 2018 and 2023.

Inclusion criteria were patients submitted to minimally invasive robot-assisted two-field Ivor-Lewis esophagectomy with intrathoracic anastomosis for oncological indications. Exclusion criteria were patients submitted to esophagectomy for non-oncological indications, patients who underwent surgery via open access (abdominal or thoracic), and patients who underwent emergency surgery.

All patients underwent preoperative diagnostic workup, including upper endoscopy with biopsy sampling to define the histologic esophageal cancer subtype and Computed Tomography (CT) scan with contrast medium to complete preoperative staging. In case of suspected advanced disease, an 18F-Fluorodeoxyglucose Positron Emission Topography-Computed Tomography (FDG PET-CT) was performed to assess metastatic spreading. The indication for optimal multimodal treatment and timing for surgery was discussed by a multidisciplinary team for each patient.

Data were collected regarding preoperative baseline patient characteristics, esophageal cancer histologic subtype and staging, neoadjuvant treatments, intraoperative and 30-day postoperative results, postoperative histologic examination, follow-up outcomes, and resource utilization. Intra-operative and postoperative complications were graded according to definitions stated by the Esophagectomy Complications Consensus Group and to the Clavien-Dindo Classification [10, 11]. Data regarding costs related to operating room time, length of stay, surgical tools, and robotic system maintenance were included.

### Surgical techniques

All patients underwent Ivor-Lewis RAMIE according to the standardized technique performed in their referral center.

H-RAMIE procedures were all performed in the same center (MM, FR), while F-RAMIE were performed in the other center by the same experienced surgeon (FB).

H-RAMIE was performed through a laparoscopic abdominal and a robot-assisted thoracoscopic approach. F-RAMIE was performed with a fully robotic approach, where both the abdominal and the thoracic phases were performed with the robotic platform. All the procedures were performed with a Xi DaVinci robotic platform with double access (thoracic and abdominal) and consisted of a subtotal esophagectomy with proximal gastrectomy, gastric tubulization and pull-up, two-field lymphadenectomy, and an intrathoracic semi-mechanic or hand-sewn esophagogastric anastomosis.

### Statistical analysis

The primary endpoint was the rate of postoperative complications of the two procedures. Secondary endpoints were perioperative (such as operative time, conversion rate, length of stay, and mortality) and follow-up outcomes, oncological results (radicality of surgical resection (R0), number of harvested lymph nodes), and overall survival.

An economic evaluation was performed from the hospital’s perspective, comparing the costs of an uncomplicated H-RAMIE and F-RAMIE case, to assess the economic sustainability of the robotic procedures. Costs related to operative room time, length of stay, laparoscopic tools (including staplers and cartridges), semi-disposable robotic surgical instruments, and robotic system maintenance were included in the analysis [12]. Costs related to the purchase of the robotic system, healthcare personnel, instrumental examinations, and complications were not considered.

Continuous variables were reported as mean ± standard deviation (DS) when normally distributed and median (Interquartile Range (IQR)) if not, while categorical data were expressed as percentages. Univariate analyses were performed to assess statistically significant differences between H-RAMIE and F-RAMIE using the t Student test for normally distributed continuous variables and the chi-square test for categorical variables.

Survival analyses were calculated using the Kaplan–Meier method and compared with the log-rank test. The follow-up period was defined for each patient as the time from the surgical procedure to the occurrence of death. The statistical significance was set to 0.05. All the statistical analyses were performed using STATA software version 18.0.

## Results

Between 2018 and 2023, a total of 100 patients underwent robot-assisted minimally invasive Ivor Lewis esophagectomy and were included in the study: 64 patients underwent H-RAMIE, while 36 patients underwent F-RAMIE at **two** different centers.

The mean age was 66.5 ± 11.2 years; 86 (86.0%) were males while 14 (14.0%) were females. The two groups had no statistically significant differences regarding age, Body Mass Index (BMI), smoking status, comorbidities, or American Society of Anesthesiologists (ASA) score. The two groups were similar in terms of cancer histotype, Siewert classification, and staging. There were more cases of patients who underwent neoadjuvant treatments in the F-RAMIE group (p < 0.001). The patients’ baseline clinical and oncological characteristics are shown in Table 1.

**Table 1:** Patients’ baseline clinical and oncological characteristics

	H-RAMIE N = 64	F-RAMIE N = 36	p-value
Age (years)	67.5 ± 11.2	64.8 ± 11.1	0.255
BMI (kg/cm^2^)	25.0 ± 4.1	25.1 ± 3.5	0.913
Gender, n (%)			
Male	56 (87.5)	30 (83.3)	
Female	8 (12.5)	6 (16.6)	0.564
Smoking, n (%) Past smoker Active smoker	28 (43.8) 15 (23.4)	13 (36.1) 6 (16.7)	0.352
Diabetes	8 (12.5%)	2 (5.6)	0.267
Cardiological comorbidities	12 (18.8)	6 (16.7)	0.795
Pneumological comorbidities	7 (10.9)	4 (11.1)	0.979
Neurological comorbidities	9 (14.1)	3 (8.3)	0.397
Vascular comorbidities	7 (10.9)	6 (16.7)	0.414
Past history of oncological disease	7 (10.9)	5 (13.9)	0.626
ASA score I II III IV	2 (3.1) 16 (25.0) 45 (70.3) 1 (1.5)	0 (0) 17 (47.2) 18 (50.0) 1 (2.8)	0.077
Cancer histotype Adenocarcinoma Squamous cell carcinoma	54 (83.4) 9 (14.1)	33 (96.7) 4 (11.1)	0.580
Siewert classification Siewert I Siewert II	28 (43.8) 36 (56.3)	13 (36.1) 23 (63.9)	0.456
Barrett associated	15 (23.4)	6 (16.7)	0.425
Staging Stage I Stage II Stage IIB Stage III Stage IVA Stage IVB	2 (3.1) 4 (6.3) 9 (14.1) 38 (59.4) 10 (15.6) 1 (1.6)	0 (0) 0 (0) 1 (2.8) 25 (69.4) 9 (25.0) 1 (2.8)	0.145
Neoadjuvant treatments	36 (56.3)	28 (77.8)	< 0.001

*H-RAMIE* Hybrid Robot-Assisted Minimally Invasive Esophagectomy, *F-RAMIE* Fully Robotic-Assisted Minimally Invasive Esophagectomy, *BMI* Body Mass Index, *ASA* American Society of Anesthesiologists

### Perioperative outcomes

The overall mean operative time was significantly longer for F-RAMIE compared to H-RAMIE (477.3 ± 47.5 vs. 434.7 ± 46.4 min, p < 0.001), and it was mainly determined by the abdominal phase, since there were no differences in terms of operative time for the thoracic phase (201.1 ± 33.4 for H-RAMIE vs. 204.9 ± 36.6 min for F-RAMIE, p = 0.597). There were five conversions to open surgery (7.8%), all in the abdominal phase, for H-RAMIE, while no conversions were required in F-RAMIE patients (p = 0.085).

All H-RAMIE patients had a semi-mechanic side-to-side esophagogastric anastomosis, while in the F-RAMIE group, 23 (63.8%) had a robot-assisted hand-sewn end-to-side and 13 (36.2%) had a semi-mechanic side-to-side esophagogastric anastomosis. There was no difference between the two groups in anastomotic leak rate but a significantly higher rate of anastomotic stenosis in the F-RAMIE group (16.6% vs. 1.6%, p = 0.026).

There were no differences in total length of stay (18.1 ± 12.6 days for H-RAMIE and 16.1 ± 10.2 days for F-RAMIE p = 0.42), but a reduced Intensive Care Unit (ICU) stay for F-RAMIE (2.3 ± 2.3 vs. 1.1 ± 1.1, p = 0.002).

According to the Clavien-Dindo classification, overall complications were similar between the two groups (45.3% in H-RAMIE and 52.7% in F-RAMIE, p = 0.53). Severe complications, defined as Clavien-Dindo ≥ 3, were 11 (17.2%) for H-RAMIE and 8 (22.2%) for F-RAMIE (p = 0.59). Table 2 summarizes postoperative complications according to the Esophagectomy Complications Consensus Group and Clavien Dindo classification [10, 11]. There was a higher rate of gastrointestinal postoperative complications in F-RAMIE, mainly caused by delayed conduit emptying. This result could be explained by the different techniques (surgical and endoscopic) of pyloroplasty in the two groups, however no data were collected regarding the type of pyloromyotomy performed.

**Table 2:** Postoperative complications according to Esophagectomy Complications Consensus Group and Clavien Dindo Classification [10, 11]

Complication, n (%)	H-RAMIE (N = 64)	F-RAMIE (N = 36)	p-value
Pulmonary Pneumonia Pleural effusion Pneumothorax Atelectasis Acute Respiratory Distress Syndrome	8 (12.5) 4 (6.3) 2 (3.1) 5 (7.8) 2 (3.1)	4 (11.1) 1 (2.8) 2 (5.6) 1 (2.8) 1 (2.8)	0.813
Cardiac, n (%) Atrial dysrhythmia Congestive heart failure	6 (9.4) 1 (1.6)	4 (11.1) 0 (0)	0.603
Infection, n (%) Wound infection Central intravenous line infection Intrathoracic/intra-abdominal abscess Generalized sepsis Other infections	2 (3.1) 2 (3.1) 2 (3.1) 2 (3.1) 4 (6.3)	0 (0) 0 (0) 4 (11.1) 1 (2.8) 0 (0)	0.149
Gastrointestinal, n (%) Feeding J-tube complications Gastrointestinal bleeding Delayed conduit emptying	2 (3.1) 1 (1.6) 1 (1.6)	4 (11.1) 0 (0) 4 (11.1)	**0.021**
Urologic, n (%) Urinary retention Urinary tract infection	4 (6.3) 1 (1.6)	0 (0) 0 (0)	0.228
Thromboembolic, n (%) Deep venous thrombosis Pulmonary embolus Peripheral thrombophlebitis	0 (0) 1 (1.6) 2 (3.1)	1(2.8) 0 (0) 0 (0)	0.324
Neurologic/Psychiatric, n (%) Acute delirium	2 (3.1)	0 (0)	0.284
Anastomotic leak, n (%) Type I Type II Type III	1 (1.6) 3 (4.7) 1 (1.6)	3 (8.3) 1(2.8) 1(2.8)	0.374
Chyle leak severity, n(%) Type IA Type IIA	1 (1.6) 0 (0)	1(2.8) 1(2.8)	0.371
Conduit necrosis/failure, n (%)	0 (0)	1 (2.8)	0.180
Recurrent laryngeal nerve injury, n (%) Type IA	0 (0)	2 (5.6)	0.163
Clavien-Dindo, n (%) I II IIIA IIIB IVA V	3 (4.7) 15 (23.4) 3 (4.7) 2 (3.1) 4 (6.25) 2 (4.7)	0 (0) 11(30.5) 3 (8.3) 2 (5.5) 3 (8.3) 0 (0)	0.624

*H-RAMIE* Hybrid Robot-Assisted Minimally Invasive Esophagectomy, *F-RAMIE* Fully Robotic-Assisted Minimally Invasive Esophagectomy

The two groups were similar regarding reinterventions (4.7% vs. 8.3%, p = 0.461), ICU readmissions (9.4% vs. 13.8%, p = 0.489), and 90-day mortality (3.1% vs. 0%, p = 0.284).

### Oncological outcomes and overall survival

The definitive histopathologic diagnosis was adenocarcinoma in 83 (83%), squamous carcinoma in 4 (4%), mixed adeno-squamous carcinoma in 2 (2%), high-grade dysplasia in 2 (2%) and no residual disease after neoadjuvant treatments in 9 (9%) patients.

The mean number of harvested lymph nodes was higher in F-RAMIE (43.8 ± 15.2 vs. 22.4 ± 10.3, p < 0.001), both in the abdominal (27.5 ± 11.1 vs. 15.6 ± 7.7, p < 0.001) and in the thoracic field (16.5 ± 9.4 vs. 7.5 ± 7.6, p < 0.001). There were no differences in terms of R0 resection (96.8% vs. 94.4% p = 0.941).

**Fig. 1:**
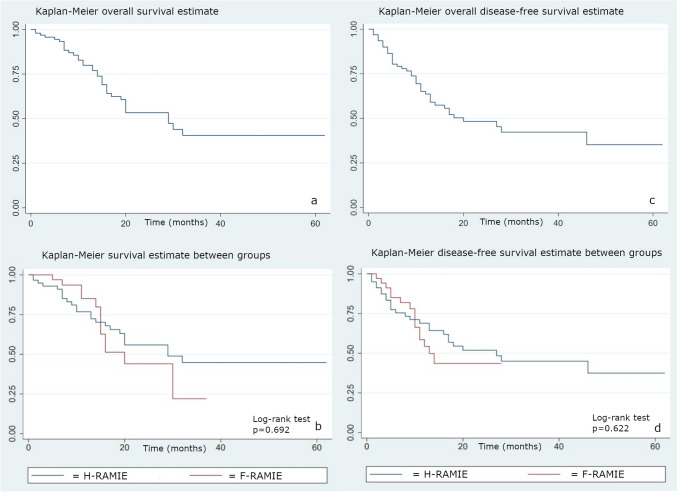
Kaplan–Meier curves for overall survival of the entire cohort (**a**) and of the two groups (**b**); Kaplan–Meier disease-free survival of the entire cohort (**c**) and of the two groups (**d**). H-RAMIE: Hybrid Robot-Assisted Minimally Invasive Esophagectomy; F-RAMIE: Fully Robotic-Assisted Minimally Invasive Esophagectomy

According to Kaplan–Meier curves, overall survival was 93.3% at 6 months, 79.9% at 12 months, and 40.6% at 24 and 48 months after surgery. The recurrence rate was 20.8% at 6 months and 51.7% at 24 months. There were no significant differences between the two groups in overall survival (p = 0.692) and time to recurrence (0.662) (Fig. 1). At univariate analysis, factors associated with worse survival were the staging of the disease (p = 0.069) and the presence of vascular invasion at postoperative definitive histopathologic examination (p = 0.017). There were no differences in survival depending on the histotype (p = 0.588) and preoperative neoadjuvant treatment (p = 0.678).

### Economic evaluation

**Fig. 2:**
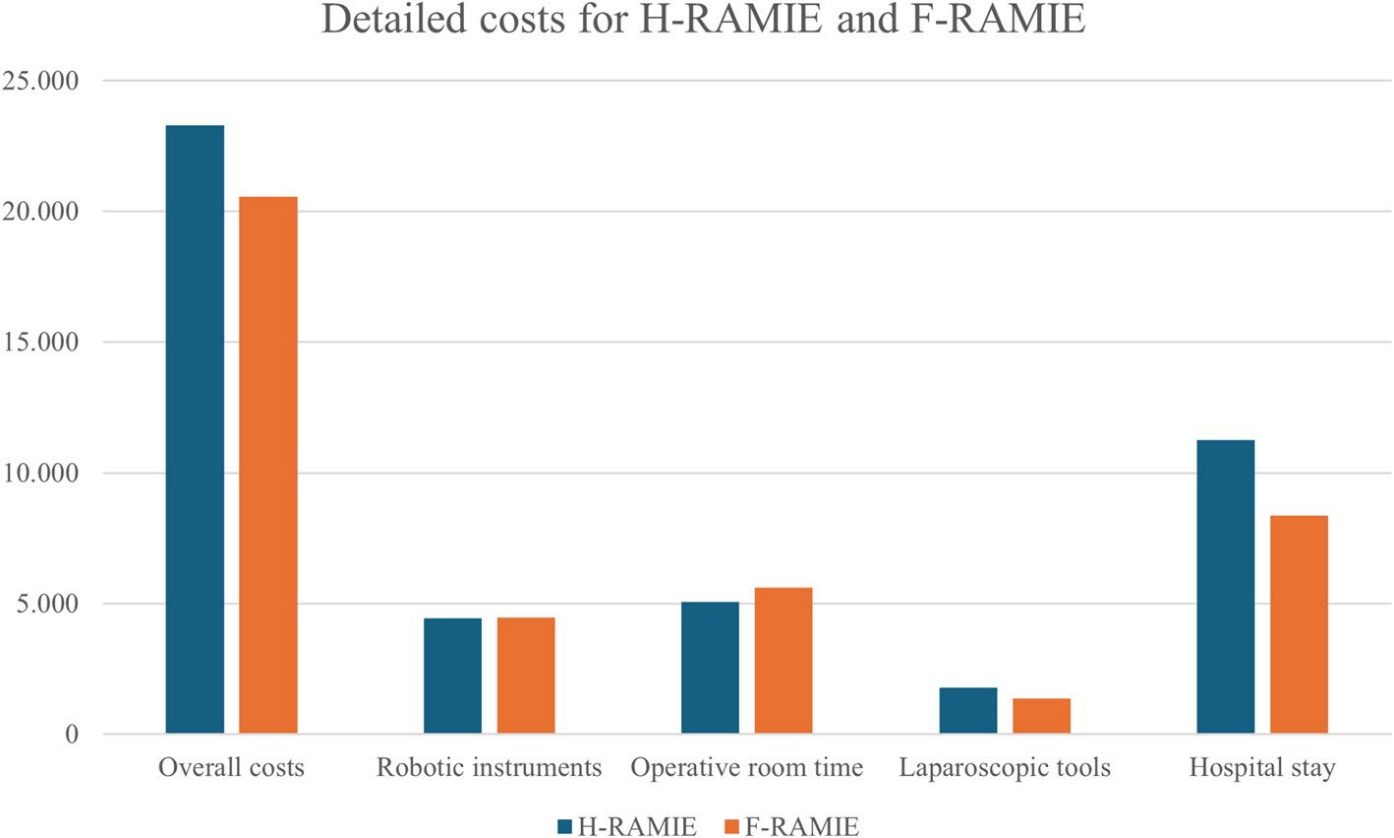
Detailed cost for H-RAMIE and F-RAMIE. *H-RAMIE* Hybrid Robot-Assisted Minimally Invasive Esophagectomy, *F-RAMIE* Fully Robotic-Assisted Minimally Invasive Esophagectomy

The overall costs were 23,302.4 ± 5,894.5 € for H-RAMIE and 20,556.3 ± 3,601.2 € for F-RAMIE (p = 0.012). The higher costs of H-RAMIE were mainly due to the costs related to hospital stay (11,267.6 ± 5,912.8 for H-RAMIE vs. 8,360.3 ± 3550.6 €, p = 0.007), while operative costs were similar between the two procedures (11,282.5 ± 574.8 € for H-RAMIE and 11,443.7 ± 1200.9 € for F-RAMIE, p = 0.36). Figure 2 illustrates the detailed costs of the two procedures.

## Discussion

Esophagectomy is the mainstay of localized esophageal cancer treatment, but this highly complex procedure is still burdened with severe morbidity and mortality [13, 14]. Therefore, less invasive approaches have been developed during the last decades to reduce surgical trauma and improve postoperative outcomes. Minimally invasive esophagectomy (MIE) has been shown to result in enhanced recovery and reduced postoperative complication rates compared to traditional open surgery, without compromising long-term survival [15, 16]. However, MIE can be technically demanding, particularly during mediastinal dissection, requiring a significant learning curve and possibly sacrificing oncological radicality, especially in the early learning stages [17, 18]. Given the favorable results of the robotic platform in other surgical fields, its application to esophageal surgery was a natural evolution [19].

Robot-assisted esophagectomy (RAMIE) was introduced to overcome the technical difficulties of conventional minimally invasive techniques, particularly in the thoracoscopic phase [6, 7]. In contrast, the technical advantages in the abdominal phase are less evident [9]. The term “RAMIE” comprises two main techniques of esophagectomy, depending on whether the robotic system is applied both in the abdominal and thoracic phases (Full-RAMIE) or only in the thoracic phase, associated with a laparoscopic or open abdominal approach (Hybrid-RAMIE).

Given that the main drawbacks generally associated with robotic surgery are longer operative times and costs, the limitation of robotic assistance to the thoracic phase has been proposed to reduce operative time and overall costs [20, 21]. On the other hand, since the robotic platform requires a considerable economic investment, optimizing the robotic system for the entire procedure could be more cost-effective. Furthermore, the reduction of costly complications could balance the high costs of the robotic system.

To date, there is a paucity of data in the literature comparing the two approaches. Grimminger et al. performed a multicentric study involving five German centers comparing F-RAMIE (175 patients) and Hybrid-RAMIE (67 patients), defined as a combination of robot-assisted thoracic and either laparoscopic or open surgical abdominal phases. This study reported a statistically significant reduction in overall postoperative morbidity (32.0% vs. 47.8%, p = 0.026), respiratory failure (1.1% vs. 7.5% p = 0.019), and anastomotic leak (10.3% vs. 22.4%, p = 0.020) for F-RAMIE group. However, these results could be biased by the learning curve effect and by the percentage of open procedures in the H-RAMIE group, considering the number of abdominal open procedures and a 13.4% conversion rate to open surgery of laparoscopic procedures [22].

Jung et al. analyzed the data of a multicenter international registry of 807 patients who underwent RAMIE. After propensity-score matching, 296 F-RAMIE patients were compared to 296 laparoscopic H-RAMIE patients. There were no differences in blood loss, operative time, radical resection rate, and number of harvested lymph nodes between the two procedures. H-RAMIE was associated with higher rates of anastomotic leak (28.0% vs. 16.6%, p = 0.001), severe complications (45.3% vs. 26.0%, p < 0.001), and length of stay (15 vs 12 days, p < 0.001) than F-RAMIE. Again, as the authors stated, these results could be biased by the learning curve effect since hybrid procedures were frequently performed at the beginning of surgeons’ robotic experience, while the shift towards F-RAMIE occurred with the increased proficiency in the use of the robotic platform [23].

The results of our study demonstrated that both procedures appear to be equally safe and feasible, with comparable outcomes in terms of postoperative morbidity. F-RAMIE was associated with longer operative time than H-RAMIE, and a significant difference in the abdominal phase mainly drove this result. This finding could be partially explained by the time spent in the “docking” of the robotic platform and instruments. Our results contrast those reported by other authors, documenting no differences or, conversely, a reduction in overall operative time for F-RAMIE compared to H-RAMIE [22, 23]. However, those findings need to be evaluated in light of a learning curve effect, while in this study, all the participating centers had extensive experience both in laparoscopic and robotic surgeries since the beginning of the study.

Our data did not show a significant difference in terms of anastomotic leaks, but a higher rate of anastomotic stricture in F-RAMIE, where an end-to-side hand-sewn robotic anastomosis was performed, which led the operator to shift toward a stapled anastomosis for the latest cases. These findings align with the results published by Kamarajah et al. in a recent network meta-analysis of anastomotic techniques for esophagectomy, including 37 randomized and non-randomized studies for a total of 8616 patients [24]. The authors reported that semi-mechanical esophagogastric anastomosis was associated with a lower anastomotic leak rate (odd ratio (OR) 0.50, 95% Confidence Interval (CI) 0.33–0.74, p = 0.001) and a lower anastomotic stricture rate (0.32, 95%CI 0.19–0.54, p < 0.001) compared to hand-sewn anastomosis. However, due to the heterogeneity of the included studies and the limited specific evidence on semi-mechanical anastomoses, other data from large multicenter international registries are advocated to provide additional evidence.

In this study, we did not show differences in terms of oncological radical resection (R0 resections) between the two procedures, but there was a higher rate of lymph node retrieval in F-RAMIE in both the thoracic and the abdominal fields. It is generally accepted that at least 15 lymph nodes should be retrieved in order to definite “adequate” a lymphadenectomy [25]. An appropriate lymphadenectomy plays an essential role in providing a correct staging of the disease, but whether it determines an improvement in overall survival is unclear [26]. In this study, both procedures demonstrated to achieve an adequate lymphadenectomy, and the number of retrieved lymph nodes was not associated with a difference in overall survival. Furthermore, it should be noted that the difference in the number of harvested lymph nodes could be explained by the extent of the surgical resection, but also by the histopathological examination, since different pathologists processed the surgical specimens.

We demonstrated in this study that F-RAMIE was associated with lower costs than H-RAMIE. Procedural costs were similar between the two groups, meaning that the costs related to the longer operative time of F-RAMIE were balanced by the increased use of laparoscopic instruments needed for H-RAMIE. According to our data, the main driver of costs was the length of stay, especially in programmed ICU, which was higher for H-RAMIE, but was not associated with significant differences in postoperative morbidity. The reduced ICU stay for F-RAMIE was not associated with increased ICU readmission or postoperative mortality.

This study has some limitations that deserve comment. The surgical procedures were not performed by the same surgeon. However, all three surgeons operate in high-volume centers and have extensive experience in laparoscopic and robotic surgery. Furthermore, the economic evaluation could be biased by the different postoperative recovery protocols of the involved centers, since the difference was mainly driven by hospital stay-related costs. The selection bias was low, as there were no selection criteria for patients to be treated in once center rather than the other, and there are less than 30 km between the two institutions, which are the main referral centers of the region. The detection bias was also low, as we adopted objectively defined primary and secondary outcomes, that could not influenced by the outcome assessor.

## Conclusions

This study shows that F-RAMIE is equally safe and effective as H-RAMIE in terms of postoperative complications and mortality, without compromising oncological radical resection rate, number of harvested lymph nodes, or long-term survival. The implementation of the robotic assistance during the abdominal phase of RAMIE appears to be sustainable from an economic perspective. Further studies comparing F-RAMIE and H-RAMIE are awaited to provide stronger evidence on the optimal application of the robotic technology during Minimally Invasive Esophagectomy.

## Data Availability

The data that support the findings of this study are available from the corresponding author upon reasonable request.
